# Changes in soil microbial biomass and organic C pools improve the sustainability of perennial grass and legume system under organic nutrient management

**DOI:** 10.3389/fmicb.2023.1173986

**Published:** 2023-04-21

**Authors:** Arvind Kumar Rai, Nirmalendu Basak, Anoop Kumar Dixit, Suchit Kumar Rai, Sanjoy Kumar Das, J. B. Singh, Sunil Kumar, T. Kiran Kumar, Priyanka Chandra, Parul Sundha, Sandeep Bedwal

**Affiliations:** ^1^ICAR–Indian Grassland and Fodder Research Institute, Jhansi, Uttar Pradesh, India; ^2^ICAR–Central Soil Salinity Research Institute, Karnal, Haryana, India; ^3^ICAR-Central Inland Fisheries Research Institute, Kolkata, West Bengal, India; ^4^ICAR–Central Tobacco Research Institute, Rajahmundry, Andhra Pradesh, India

**Keywords:** carbon input, dissolved organic carbon, microbial biomass C, semi-arid agroecosystem, SOC indices, SOC sequestration, stratification ratio

## Abstract

**Introduction:**

The perennial grass–legume cropping system benefits soil because of its high biomass turnover, cover cropping nature, and different foraging behaviors. We investigated the response of soil organic carbon (SOC) pools and their stock to organic and inorganic nutrient management in the Guinea grass and legume (cowpea-Egyptian clover) cropping system.

**Methods:**

Depth-wise soil samples were collected after harvesting the Egyptian clover. Based on the ease of oxidation with chromic acid, different pools of SOC oxidizable using the Walkley–Black C method, very labile, labile, less labile, non-labile; and dissolved organic C (DOC), microbial biomass C (MBC), and total organic C (TOC) in soils were analyzed for computing several indices of SOC.

**Result and discussion:**

After 10 years of crop cycles, FYM and NPKF nutrient management recorded greater DOC, MBC, SOC stocks, and C sequestration than the NPK. Stocks of all SOC pools and carbon management index (CMI) decreased with soil depth. A significant improvement in CMI, stratification ratio, sensitivity indices, and sustainable yield index was observed under FYM and NPKF. This grass–legume intercropping system maintained a positive carbon balance sequestered at about 0.8Mg C ha^−1^ after 10 years without any external input. Approximately 44–51% of the applied carbon through manure was stabilized with SOC under this cropping system. The DOC, MBC, and SOC in passive pools were identified for predicting dry fodder yield. This study concludes that the application of organics in the perennial grass–legume inter cropping system can maintain long-term sustainability, enhance the C sequestration, and offset the carbon footprint of the farm enterprises.

## Graphical Abstract

Nutrient management affects biomass production and SOC sequestration. 
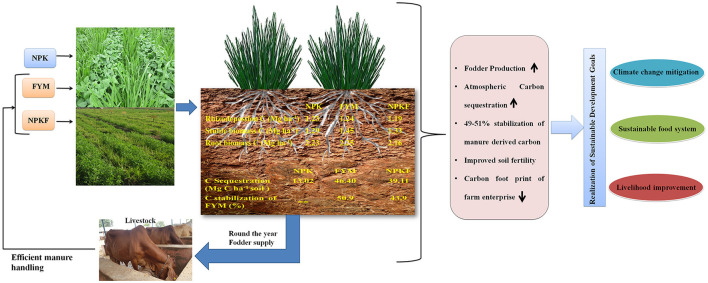

## 1. Introduction

Soil organic carbon (SOC) is key to soil health management influencing crop production, climate change mitigation, and other ecosystem services (Srinivasarao et al., 2016; Oldfield et al., 2019; Hasegawa et al., 2021; Doblas-Rodrigo et al., 2022). The accrual and hoarding of SOC are determined greatly by the set of biophysical variables such as temperature, rainfall, potential evapotranspiration, soilscape, physiography, vegetation, cropping systems, and management practices (Schmidt et al., 2011; Lal, 2015; Poffenbarger et al., 2018; Alavi-Murillo et al., 2022). The cropping systems and associated management practices utilizing inorganic fertilizer and organic manures alone or in combination are the key drivers influencing the SOC stock of soil in a given agroecosystem (Mandal et al., 2008; Ghosh et al., 2012; Luo et al., 2014; Sainju et al., 2014; Yadav et al., 2017; Poffenbarger et al., 2018; Ngangom et al., 2020; Basak et al., 2021a). These management practices affect the separation of SOC in pools of varied stability (Basak et al., 2021a) characterized by the susceptibility to oxidation and residence period (Zimmermann et al., 2007). A faster turnover rate of labile C fraction of SOC (Sanderman et al., 2017) and its oxidation maintain the productivity of soil and CO_2_ flux to the atmosphere. Conversely, the passive pool has a long residence time because of physical and biochemical protection, making it resistant to microbial actions (Zimmermann et al., 2007; Weil et al., 2017; Datta et al., 2018). Previous studies reported the effect of the cropping system on the distribution of active and passive pools in soils of subtropic (Chaudhary et al., 2017; Yadav et al., 2017, 2019; Anantha et al., 2020; Babu et al., 2020; Basak et al., 2021a), arid (Moharana et al., 2017), and semi-arid (Srinivasarao et al., 2013, 2016; Mitran et al., 2018; Basak et al., 2021b) regions. The application of organic fertilizers is also reported to improve the yield of grain crops through their influence on SOC and associated soil properties (Mandal et al., 2007). However, similar studies involving perennial grass-based intensive forage production in semi-arid climatic conditions are lacking (Ghosh et al., 2010; Kim et al., 2022).

In India, 57% of the net sown areas are rainfed under different agroecosystems (Bal et al., 2022). A significant proportion of the area under semi-arid regions in India had been brought under canal irrigation by developing dams on the rivers with seasonal water flow. As a result, cropping intensity in these canal commands increased. The limited supply of canal water only during the winter makes it a rainfed (monsoon) rotation followed by irrigated (winter) crops in these regions. Guinea grass (*Panicum maximum* cv. Hamil) intercropped with seasonal legumes is an important intensive forage production system in rainfed-irrigated areas in semi-arid regions of India (Kumar et al., 2012). Fast growth and high biomass production make it an ideal crop for fodder and biofuel production (Mohapatra et al., 2019) and SOC sequestration in pastures and grassland (Ghosh et al., 2009; Sarkar et al., 2020). Cowpea and Egyptian clover (*Trifolium alexandrinum* L) are leguminous crops commonly incorporated in grass-based cropping systems to improve forage quality and enhance forage productivity during the poor growth of grass components (Kumar et al., 2012). The cowpea and Egyptian clover also complement the cropping system's soil SOC pools and productivity because of spatial and temporal annidation and different rooting behaviors (Rai et al., 2013).

The SOC stock and stability indices developed from SOC pools were found better in assessing the impact of management practices and cropping systems in several cropping systems in different agro-ecologies (Blair et al., 1995; Franzluebbers, 2002; Mandal et al., 2008). But, there is limited information on the perennial grass + legumes-based intensive intercropping system for biomass production under inorganic and organic nutrition with rainfed-irrigated water management. These indices in a perennial grass–legume-based intensive cropping system will capture the impact of different nutrient management on SOC and their interactions with soil properties in rainfed-irrigated agroecosystems of the semi-arid region. This information is essential from an agronomic and environmental point of view for crop planning and carbon budgeting at the farm scale. Therefore, this study was conducted to assess the impact of the organic and inorganic nutrients alone or in combination with the stock and stability of SOC in perennial guinea grass + the (cowpea-Egyptian clover) cropping system under rainfed (monsoon)—irrigated (winter) water management. The objective of the study was to (i) assess the impacts of organic, inorganic, and integrated nutrient management on depth-wise SOC stock and its sequestration potential; (ii) measure the effect of nutrient management on the pool-wise allocation of SOC of different stability, and (iii) to establish a relationship between SOC pools and cropping system sustainability.

## 2. Materials and methods

### 2.1. Experimental site

This experiment was conducted in the semi-arid, continental, and monsoonal climate at the Central Research Farm (25°31′ 01.73″ N, 78°33′ 32.84″ E, at 224 m mean sea level), ICAR- Indian Grassland and Fodder Research Institute, Jhansi, Uttar Pradesh, India. The region's annual rainfall is 906.5 mm (781 mm during the rainy season and 52 mm during the winter), with an annual potential evapotranspiration of 1,512 mm. The soil of the experimental site is classified under hyperthermic *Typic Haplustepts* with silty loam in texture (sand, silt, and clay with 36.6, 43.2, and 20.2%, respectively). The soil of the experimental site had pH_1:2_ of 7.9 and Walkley–Black organic carbon (Walkley and Black, 1934) of 4.2 g kg^−1^; and KMnO_4_ oxidizable N (Subbiah and Asija, 1956), Olsen's extractable P (Jackson, 1967), and ammonium acetate extractable K (Jackson, 1967) of 192, 9.9, and 135 kg ha^−1^, respectively.

### 2.2. Field experiment: treatment detail and layout

The field experiment was conducted following a randomized complete block design with three replications. The fertilizers treatments were as follows: control, the recommended dose of N, P, and K (NPK) fertilizers, farmyard manure (FYM), and integrated application of FYM and NPK (NPKF) were allocated randomly in triplicate in plots of 4 × 5 m^2^ separated by 1-meter buffer strip from all sides to prevent treatment interference. Details of the treatments imposed, including source, application schedule, and other cultural practices followed, are given in Table 1. The first crop Guinea grass (*Panicum maximum* cv. Hamil) rooted slips were planted in July 2004 at a 100 × 50 cm distance. Cowpea (*Vigna unguiculata cv*. EC-4216) and Egyptian clover (*Trifolium alexandrinum cv*. Wardan) were planted in the inter-row space in summer (June–July) and winter season (November–March), respectively. In their respective season, two rows of cowpea and berseem were planted in the inter-row space at a 30 cm distance. In FYM and NPKF treatments, FYM was applied in the rainy (July–October) and winter (November–June) seasons on a fresh weight basis with an average moisture content of 35% (w/w). The FYM contained ~8.7 ± 0.4, 2.0 ± 0.3, and 12.1 ± 0.2 g kg^−1^ of N, P, and K, respectively. The mean C: N ratio of the manure was 70:1. Rainy (July-October)-season crop was grown rainfed, while in winter season 7–8, irrigation was applied depending upon the winter rains and the crop's need in the cropping system. The above-ground biomass of the Egyptian clover was harvested at 30-day intervals, while cowpea was harvested 60 days after sowing. Guinea grass was harvested 5–7 cm above the ground surface at 30 and 60 days intervals in rainy (July–October) and the rest of the season (November–June), respectively.

**Table 1:** Description of the management practices for guinea grass + (cowpea-Egyptian clover) cropping system.

**Treatments detail**		**Nutrient management**		**Tillage**
		**Rainy season (July-October)**	**Winter season (November–February)**	
Control	Unfertilized control (without NPK or organics)	-	-	One harrowing followed by leveling and line sowing of cowpea and Egyptian clover at 30 cm spacing in rainy and winter seasons respectively
NPK	100% RDF (recommended dose of fertilizer (N: P : K kg ha^−1^)	200:50:50	20:80:0	
FYM	Farmyard manure (60.0 Mg ha^−1^)	37.5	22.5	
NPKF	FYM (52.5) + 25% RDF in rainy season	37.5 Mg ha^−1^ + (50:12.5:12.5 kg ha^−1^)	15.0 Mg ha^−1^	

Fertilizers N, P, and K were applied in the form of urea, single super phosphate, and muriate of potash, respectively.

### 2.3. Soil analysis

Soil samples were collected randomly from three representative sites in each plot at the field moist condition from four depths *viz*., 0–0.15, 0.15–0.30, 0.30–0.50, and 0.50–0.70 m after harvesting the Egyptian clover in the last week of April 2014. One set of soil samples was immediately preserved at 4°C for microbial biomass carbon (MBC) estimation (Vance et al., 1987). Another sample set was air-dried and processed to pass through a 2 mm sieve for further analysis of SOC pools. Depth-wise bulk density of undisturbed soil was measured using a core sampler (Blake and Hartage, 1986). A pH meter (Eutech pH 700) was used for estimating soil pH in a 1:2 soil: water suspension (Jackson, 1967). Soil texture (Gee and Bauder, 1986) and calcium carbonate (Allison and Moodie, 1965) were determined using standard procedure. Total carbon (TC) was estimated using CHNS Elemental Analyzer (Vario EL III, Germany). The TOC was computed by deducting the carbon derived from calcium carbonate (Allison and Moodie, 1965) from TC.

#### 2.3.1. Microbial biomass carbon, microbial quotient, and dissolved organic carbon

Chloroform fumigation and extraction method were used to determine microbial biomass carbon (MBC) in fresh soil samples (Vance et al., 1987). The difference in the C flux from fumigated to unfumigated samples was used to calculate the MBC (Mg ha^−1^) using equation (1) proposed by Voroney and Paul (1984). The dissolved C in the extract was determined using the method of McGill et al. (1986). The microbial quotient (MQ) was calculated from MBC and TOC (Equation 2).


(1) $$\begin{array}{l} MBC=\;\frac{Microbial\;carbon\;flush}{0.41} \end{array}$$

(2) $$\begin{array}{l} MQ\;\left(\%\right)\;=\;\frac{MBC}{TOC}\;\times 100 \end{array}$$

#### 2.3.2. Oxidizable soil organic carbon and its pools

The SOC pools were determined using the modified method suggested by Chan et al. (2001). Briefly, soils were treated with 12, 18, and 24 *N* H_2_SO_4_ and 0.5 *N* K_2_Cr_2_O_7_. The obtained TOC was separated into four pools of reduced oxidability: very labile (C_VL_), labile (C_L_), less labile (C_LL_), and non-labile (C_NL_) following the procedure described in Majumder et al. (2007). C_VL_ + C_L_ and C_LL_ + C_NL_ were expressed as the active (C_AP_) and passive pools (C_PP_), respectively.

### 2.4. Crop-derived C inputs into the soil

After harvesting multi-cut guinea grass, cowpea, and Egyptian clover, the leftover biomass and root samples were collected. The protocol described by Thangaraj and O'Toole (1986) was used to sample guinea grass, cowpea, and Egyptian clover roots. The C content of these samples was analyzed. The net rhizodeposition of grass and legume crops was derived from roots' biomass, as described by Pausch and Kuzyakov (2018). A detailed assessment was made for crop residue C added from guinea grass, cowpea, and Egyptian clover. Total carbon input to the soil from rhizodeposition, roots, stubbles, and leaves of the component crops was measured annually during the last 3 years using respective biomass addition and carbon content.

### 2.5. Soil carbon stock

The soil carbon stock (Mg ha^−1^) was calculated using bulk density (Mg m^−3^), depth of soil layer (m), and C content (g C g^−1^) in the respective soil layer using Equation (3) (Batjes, 1996).


(3) $$\begin{array}{l} Carbon\;stock\;in\;soil\;=\left(Carbon\;content\right)\;\times Bulk\;density\\ \times \;Depth \end{array}$$

### 2.6. Recalcitrant index

To assess the effect of FYM, inorganic fertilizer, and control, the recalcitrant index (RI_1_ and RI_2_) of SOC was calculated using Equations (4) and (5) (Datta et al., 2018). The RI_mean_ was computed by averaging the RI_1_ and RI_2_.


(4) $$\begin{array}{l} RI_{1}=\frac{C_{LL}+C_{NL}}{C_{VL}+C_{L}} \end{array}$$

and


(5) $$\begin{array}{l} RI_{2}=\frac{C_{NL}}{TOC} \end{array}$$

### 2.7. Carbon lability index

The carbon lability index (CLI) of SOC was developed using SOC pools of comparative oxidability using Equation (6) (Majumder et al., 2007).


(6) $$\begin{array}{l} CLI=\frac{C_{VL}}{TOC}\;\times 3+\frac{C_{L}}{TOC}\times 2+\frac{C_{LL}}{TOC}\times 1 \end{array}$$

### 2.8. Carbon management index

The CMI highlights the management-induced changes in soil quality (Basak et al., 2021a). It is derived from the carbon pool index (CPI) and lability index (LI) as shown in the following equations (Blair et al., 1995):


(7) $$\begin{array}{l} CMI=\;CPI\times LI\;\times 100 \end{array}$$

(8) $$\begin{array}{l} CPI=\frac{TOC\;\left(NPK/FYM/NPKF\right)}{TOC\;\left(unamended\;control\right)} \end{array}$$

(9) $$\begin{array}{l} LI=\frac{LC\;\left(NPK/FYM/NPKF\right)}{LC\;\left(unamended\;control\right)} \end{array}$$

(10) $$\begin{array}{l} Lability\;of\;C\;\left(LC\right)\;=\frac{C_{VL}}{TOC-\;C_{VL}} \end{array}$$

### 2.9. Carbon build-up and sequestration

The total carbon addition in the soil through organic manure was calculated based on oven-dry weight. Carbon budgeting was done for 0–0.70 m depth. The percent C build-up over control, C build-up rate, per cent stabilization of applied carbon, carbon sequestration, and carbon sequestration potential for different nutrient management strategies were estimated using the following equations as described by Mishra et al. (2015):


(11) $$\begin{array}{l} \%\;C\;build-up=\frac{\left[{TOC\;}_{Treatment\;}-{TOC\;}_{Control}\right]}{TOC_{Control}}\times 100 \end{array}$$

(12) $$\begin{array}{l} C\;build-up\;rate\;\left(Mg\;C\;ha^{1}\;yr^{-1}\right)=\frac{\left[TOC_{Treatment}-TOC_{Control}\right]}{Year\;of\;experiment} \end{array}$$

(13) $$\begin{array}{l} Percent\;stabilization\;of\;applied\;C=\frac{\left[C_{Trt}-C_{background}\right]}{C_{applied\;through\;manure}}\times 100 \end{array}$$

Here, C_Trt_ is the TOC in a particular treatment; TOC in control treatment was considered a background contribution of TOC for FYM treatment; while for NPKF treatment considering the additive effect, *C*_*control*_ + 0.25 (*C*_*NPK*−_ *C*_*control*_) was considered as the background contribution to soil TOC,


(14) $$\begin{array}{l} C\;sequestration\;\left(Mg\;C\;ha^{1}\right)=TOC_{Current}-TOC_{Initial} \end{array}$$

### 2.10. Stratification ratio

The stratification ratio (SR) specifies the sub-surface influence of surface-imposed management practices. The SR of SOC and its pools was derived as the ratio of respective carbon fractions using the following equation described by Franzluebbers (2002):


(15) $$\begin{array}{l} SR=\frac{TOC/WBOC/C_{VL}/C_{AP\;in\;surface\;soil\;\left(0-15\;cm\right)}}{TOC/WBOC/C_{VL}/C_{AP\;in\;sub-surface\;soil\;\left(15-30\;cm\right)}} \end{array}$$

The average stratification ratio of all the carbon fractions was denoted as SR_mean_.

### 2.11. Sensitivity index

The SI (%) of 0–0.15 m soil layer was calculated to assess the changes in different C pools in treated soils in comparison to control using Equation (16) proposed by Banger et al. (2010) as follows:


(16) $$\begin{array}{l} SI=\frac{\left\{TOC/WBOC/C_{VL}/CAP\;\left(NPK/FYM/NPKF\right)-TOC/WBOC/C_{VL}/CAP\;\left(unamended\;control\right)\right\}}{TOC/WBOC/C_{VL}/CAP\;\left(unamended\;control\right)}\times 100 \end{array}$$

The average SI of all the carbon fractions was denoted as SI_mean_ for the respective treatments.

### 2.12. Biometric observation and biomass yield

Total green biomass yield and dry biomass yield (oven-dry weight basis at 60°C) of the guinea grass, cowpea, and Egyptian clover were recorded plot-wise during 2004–2014. The sustainable yield index (SYI) was calculated using dry biomass yield using the following formula (Singh et al., 1990):


(17) $$\begin{array}{l} Sustainable\;yield\;index\;\left(SYI\;\right)=\\ \frac{{\left[\left(Mean\;dry\;biomass\;yield\;of\;guinea\;grass,\;cowpea\;and\;Egyptian\;clover\;during\;the\;ten\;year\right)\;-\;\sigma \right]}^{\prime }}{Maximum\;dry\;biomass\;yield\;of\;guinea\;grass,\;cowpea\;and\;Egyptian\;clover\;during\;the\;ten\;year} \end{array}$$

Where σ estimated the standard deviation in the experiment during the 10 years of cultivation.

### 2.13. Statistical analysis

All the data were tested for normality and homogeneity of variance using the Bartlett and Shapiro–Wilk tests, respectively. Analysis of variance (ANOVA) was carried out using SPSS statistical software (SPSS Inc., 2004, Chicago, USA). The pairwise comparison of the means of all the parameters was performed using Duncan's multiple range test (*P* < 0.05). The relationship between the soil parameter dry fodder yield and sustainable yield index was determined using Pearson's correlation coefficient. Multiple linear regression models were developed to establish a relation between the yield and SYI with different response variables.

## 3. Results

### 3.1. Nutrient management and SOC pools

Farmyard manure (FYM), when applied alone or in conjunction with chemical fertilizer (NPKF), increased the soil pH and decreased bulk density (BD) compared to the unamended control and sole application of NPK (Table 2). The soil pH and BD increased with soil depth. FYM had a more pronounced (*P* < 0.05) effect on the increase in Walkley–Black organic carbon (WBOC), TOC stocks, and dissolved organic C (DOC). The NPKF had greater WBOC and TOC stocks than NPK and control. The WBOC, TOC, and DOC stocks decreased at a lower depth.

**Table 2:** Changes in soil physicochemical properties with nutrient management (mean over depths) and soil depth (mean over treatments).

**Treatment**	**pH_1:2_**	**BD (Mg m^−3^)**	**WBOC (Mg ha^−1^)**	**TOC (Mg ha^−1^)**	**DOC (Mg ha^−1^)**
Control	7.65^B^	1.38^AB^	5.6^D^	14.7^C^	0.23^C^
NPK	7.40^C^	1.39^A^	7.8^C^	17.7^B^	0.31^A^
FYM	7.90^A^	1.35^C^	10.4^A^	26.1^A^	0.31^A^
NPKF	7.85^A^	1.37^B^	8.8^B^	24.8^A^	0.27^B^
Depth (m)					
0–0.15	7.38^c^	1.34^d^	10.2^a^	23.5^a^	0.32^a^
0.15–0.30	7.72^b^	1.36^c^	7.6^b^	21.4^b^	0.25^c^
0.30–0.50	7.79^b^	1.39^b^	8.1^b^	20.5^b^	0.28^b^
0.50–0.70	7.90^a^	1.41^a^	6.7^c^	17.9^c^	0.27^bc^
0–0.7	7.96	1.42	32.6	81.4	1.12
Treatment × Depth	^*^	ns	^***^	^***^	ns

Soil pH, (soil: water, 1:2 ratio); BD, bulk density; WBOC, Walkley–Black organic carbon; TOC, total organic carbon; DOC, dissolved organic C; uppercase for nutrient management and lowercase for depths within a column differ significantly by Duncan's multiple range test (*P* < 0.05); ns,

* and *** represent non-significant and significance at *P* < 0.05 and 0.001 for interaction effect (treatment × depth) in experimental sites with nutrient management (mean over depths) and soil depth (mean over treatments).

**Figure 1:**
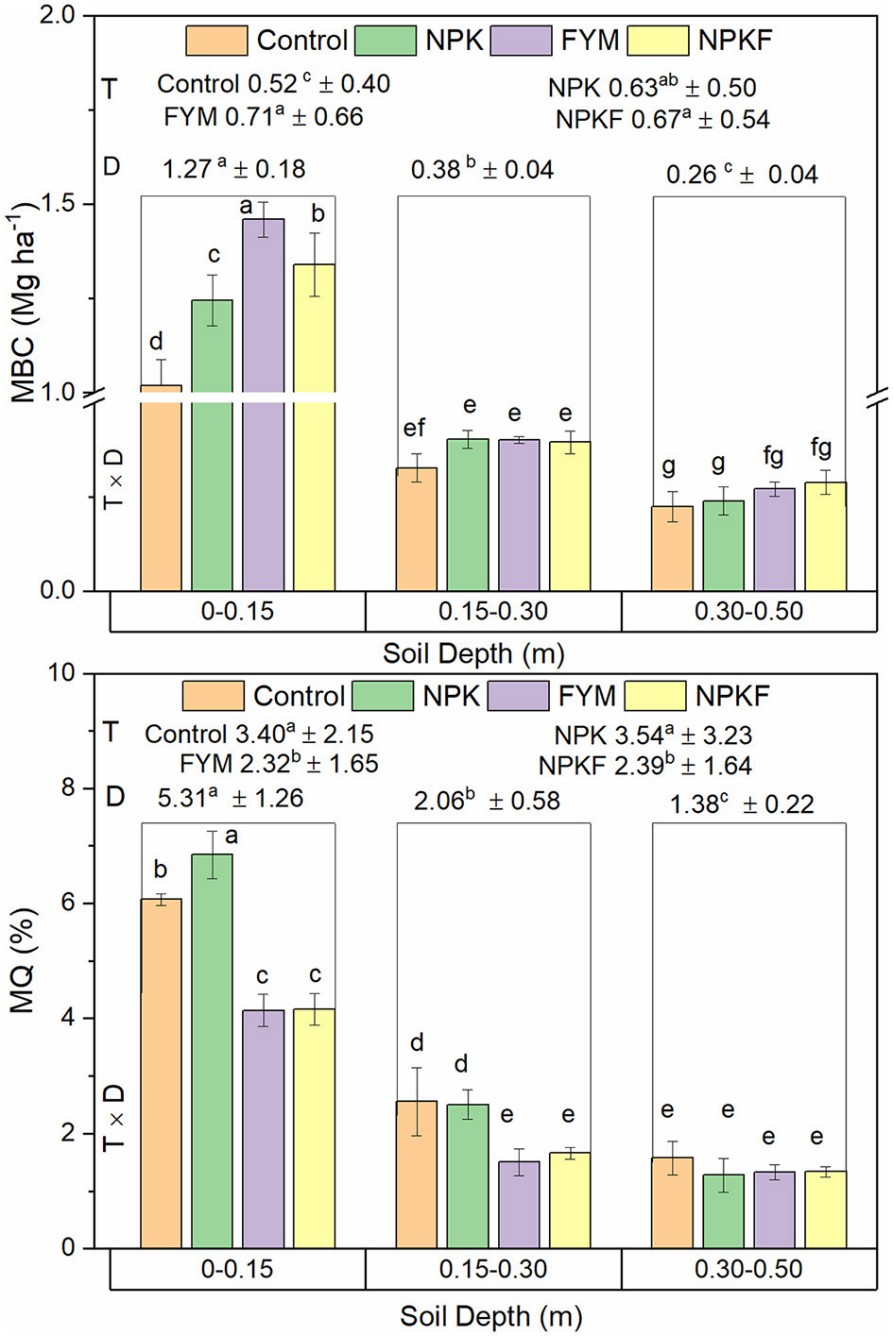
Effect of nutrient management (T), depth (D), and their interaction (T × D) on microbial biomass carbon and microbial quotient; capped lines on bars are standard deviations; *n* = 9, 12, and 3 for T, D, and T × D, respectively; numbers and bars followed by different letters (a–f) are significantly different (*P* < 0.05).

Carbon allocation in different pools varied with nutrient management (NM) strategies and soil depth (Table 3). Very labile (C_VL_), labile (C_L_), and non-labile (C_NL_) SOC stocks in FYM and NPKF were greater than unamended control and NPK. However, the less labile (C_LL_) SOC pool was greater in NPK than in NPKF and control. Irrespective of NM, approximately 25 and 37% of TOC in 0–0.70 m depth were allocated in C_L_ and C_VL_ pools, respectively (Supplementary Table S1). The per cent of C_VL_ to TOC stock remained at par for all treatments, but C_L_ and C_LL_ proportion to TOC stock was greater for NPKF and NPK, respectively. The proportion of C_L_ to TOC increased with soil depth; however, at the 0.50–0.70 m depth, the proportion of C_NL_ to TOC was greater than the upper soil layers. The active (C_AP_) and passive (C_PP_) pools constitute 61 and 42% of the TOC in 0.70 m soil depth, respectively. The FYM and NPK maintained relatively higher proportions of active and passive pools, respectively. The MBC was 8 and 37% greater for FYM and NPKF compared to NPK and control. On the contrary, microbial quotient (MQ) decreased under FYM and NPKF compared to NPK and control. Both MBC stocks and MQ decreased with an increase in soil depth (Figure 1).

**Table 3:** Changes in soil organic C pools (Mg ha^−1^) with nutrient management in soil depth.

**Treatment**	**C_VL_**	**C_L_**	**C_LL_**	**C_NL_**	**C_AP_**	**C_PP_**
Control	5.11^C^	3.68^B^	2.49^C^	3.42^B^	8.80^C^	5.90^B^
NPK	6.00^C^	3.33^B^	4.05^A^	4.30^B^	9.33^C^	8.35^A^
FYM	10.01^A^	6.30^A^	3.62^AB^	6.20^A^	16.31^A^	9.83^A^
NPKF	8.93^B^	6.33^A^	3.34^B^	6.23^A^	15.26^B^	9.57^A^
**Depth (m)**						
0–0.15	9.78^a^	3.54^b^	3.48^a^	6.73^a^	13.32^a^	10.21^a^
0.15–0.30	7.77^b^	5.80^a^	3.15^a^	4.67^b^	13.57^a^	7.82^b^
0.30–0.50	6.80^b^	5.98^a^	3.22^a^	4.54^b^	12.78^a^	7.76^b^
0.50–0.70	5.72^c^	4.31^b^	3.66^a^	4.21^b^	10.04^b^	7.87^b^
0–0.7	30.1	19.6	13.5	20.2	49.7	33.7
Treatment × Depth	^***^	^***^	^***^	ns	^***^	ns

C_VL_, very labile; C_L_, labile; C_LL_, less labile; C_NL_, non-labile; C_AP_, active; C_PP_, passive pool of soil organic carbon; uppercase for nutrient management and lowercase for depths within a column differ significantly by Duncan's multiple range test (*P* < 0.05); ns and

*** represent non-significant and significance at *P* < 0.001 for interaction effect (treatment × depth) in experimental sites with nutrient management (mean over depths) and soil depth (mean over treatments).

### 3.2. Lability index, recalcitrant index, stratification ratio, carbon management index, and sensitivity index

A lower lability index (LI) and higher recalcitrant index (RI_mean_) appeared for NPK compared to FYM (Table 4). Soils of lower depth had low LI and higher RI_mean_ values. A significant improvement in the carbon management index (CMI) was observed for FYM and NPKF compared to NPK and control (Table 4). The values of CMI decreased at lower soil depths. The stratification ratio (SR) of C_VL_ was greater under FYM and NPKF. Except for active pools, the stratification ratio of different pools was greater for FYM. The sensitivity index for different pools was also greater for FYM (Table 5).

**Table 4:** Effect of nutrient management practices on lability index (LI), recalcitrant indices (RI), and carbon management index (CMI) in soil depth.

**Treatment**	**LI**	**RI_1_**	**RI_2_**	**RI_mean_**	**CMI (%)**
Control	1.76^AB^	0.69^B^	0.23^A^	0.46^B^	57.2^B^
NPK	1.66^B^	0.91^A^	0.24^A^	0.58^A^	66.8^B^
FYM	1.85^A^	0.61^B^	0.24^A^	0.42^B^	116.5^A^
NPKF	1.81^AB^	0.65^B^	0.26^A^	0.46^B^	99.6^A^
**Depth (m)**					
0–0.15	1.57^c^	0.82^a^	0.27^a^	0.54^a^	95.1^ab^
0.15–0.30	1.86^ab^	0.61^b^	0.23^a^	0.42^b^	99.9^a^
0.30–0.50	1.91^a^	0.64^b^	0.24^a^	0.44^ab^	77.4^bc^
0.50–0.70	1.74^b^	0.80^a^	0.25^a^	0.52^ab^	67.9^c^
0–0.7	1.75	0.70	0.25	0.47	83.9
Treatment × Depth	^*^	ns	ns	ns	ns

Uppercase for nutrient management and lowercase for depths within a column differ significantly by Duncan's multiple range test (*P* < 0.05); ns and ^*^represent non-significant and significance at *P* < 0.05 for interaction effect (treatment × depth) in experimental sites with nutrient management (mean over depths) and soil depth (mean over treatments).

**Table 5:** Effect of nutrient management practices on SOC stratification ratio (SR) and sensitivity indices (SI, %).

**Treatment**	**SR_TOC_**	**SR_VL_**	**SR_AP_**	**SI_WBOC_**	**SI_TOC_**	**SI_VL_**	**SI_AP_**	**SImean**
Control	1.31^A^	1.18^AB^	1.22^A^					
NPK	1.14^A^	1.07^B^	1.04^AB^	0.32^B^	0.13^B^	0.69^B^	0.51^B^	0.41^B^
FYM	1.32^A^	1.35^A^	0.89^B^	81.63^A^	114.7^A^	95.1^A^	54.8^A^	86.5^A^
NPKF	1.36^A^	1.35^A^	0.97^B^	0.36^B^	0.36^B^	0.44^B^	0.25^B^	0.35^B^

ns, ^*^, ^**^ and ^***^represent non-significant, significance at *P* < 0.05, 0.01, and 0.001; values followed by different letters (uppercase for different treatments) within a column differ significantly by Duncan's multiple range test (*P* < 0.05).

### 3.3. Fodder yields, C input, and its sequestration and stabilization

The dry forage yield and SYI were greater than FYM (Table 6). The annual return of C through crop residues of guinea grass, cowpea, and Egyptian clover in the form of root, stubble, and rhizodeposition was estimated for different NM practices for the entire 10 years of the cropping cycle (Supplementary Table S1). The return of root, stubble, and rhizodeposition C was greater when NM practices were adopted (NPK/FYM/NPKF) than in control. Besides, crop residue C return, a 4.0 and 3.46 Mg ha^−1^ C year^−1^ are supplied through organic amendments FYM and NPKF treatment, respectively. The annual total C turnover, TOC stock, C builds-up per cent, C build-up rates, and C sequestration rate were greater with FYM (Table 6). The conspicuously cropping system alone, without any external nutrient supply, maintained a positive carbon balance and sequestrated 0.8 Mg C ha^−1^. Approximately 50.9 and 43.9% of the C applied through manure were stabilized with SOC under FYM and NPKF, respectively. The remaining 49.1 to 56.1% of applied carbon escaped from the soil.

**Table 6:** Annual return of crop residue C (Mg ha^−1^), carbon sequestration potential indices for soil (0–0.7 m), yield, and sustainable yield index for different nutrient managements in perennial grass–legume system.

**Treatment**	**Root biomass C**	**Stuble biomass C**	**Rhizodeposition C**	**Organic amendment C**	**Annual C input to the soil**	**TOC stock (Mg ha^−1^**	**% C build-up over control**	**C build-up rate (Mg C ha^−1^ y^−1^)**	**C sequestration (Mg C ha^−1^ soil)**	**Percent SOC stabilization**	**Dry forage yield (Mg ha^−1^)**	**Sustainable yield index**
Initial						56.5						
Control	1.92^B^	0.89^C^	1.06^B^	–	3.87^D^	57.36^B^			0.82^B^		220.5^C^	0.53^C^
NPK	2.23^A^	1.29^B^	1.23^A^	–	4.74^C^	69.56^B^	21.28^B^	1.22^B^	13.02^B^		302.2^AB^	0.56^B^
FYM	2.25^A^	1.45^A^	1.24^A^	4.00^A^	8.94^A^	102.94^A^	80.14^A^	4.56^A^	46.40^A^	50.9	309.2^A^	0.63^A^
NPKF	2.16^A^	1.33^AB^	1.19^A^	3.46^B^	8.15^B^	95.65^A^	66.93^A^	3.83^A^	39.11^A^	43.9	299.4^B^	0.57^B^

Values followed by different letters (uppercase for different treatments) within a column differ significantly by Duncan's multiple range test (*P* < 0.05).

### 3.4. Soil properties and system productivity

**Figure 2:**
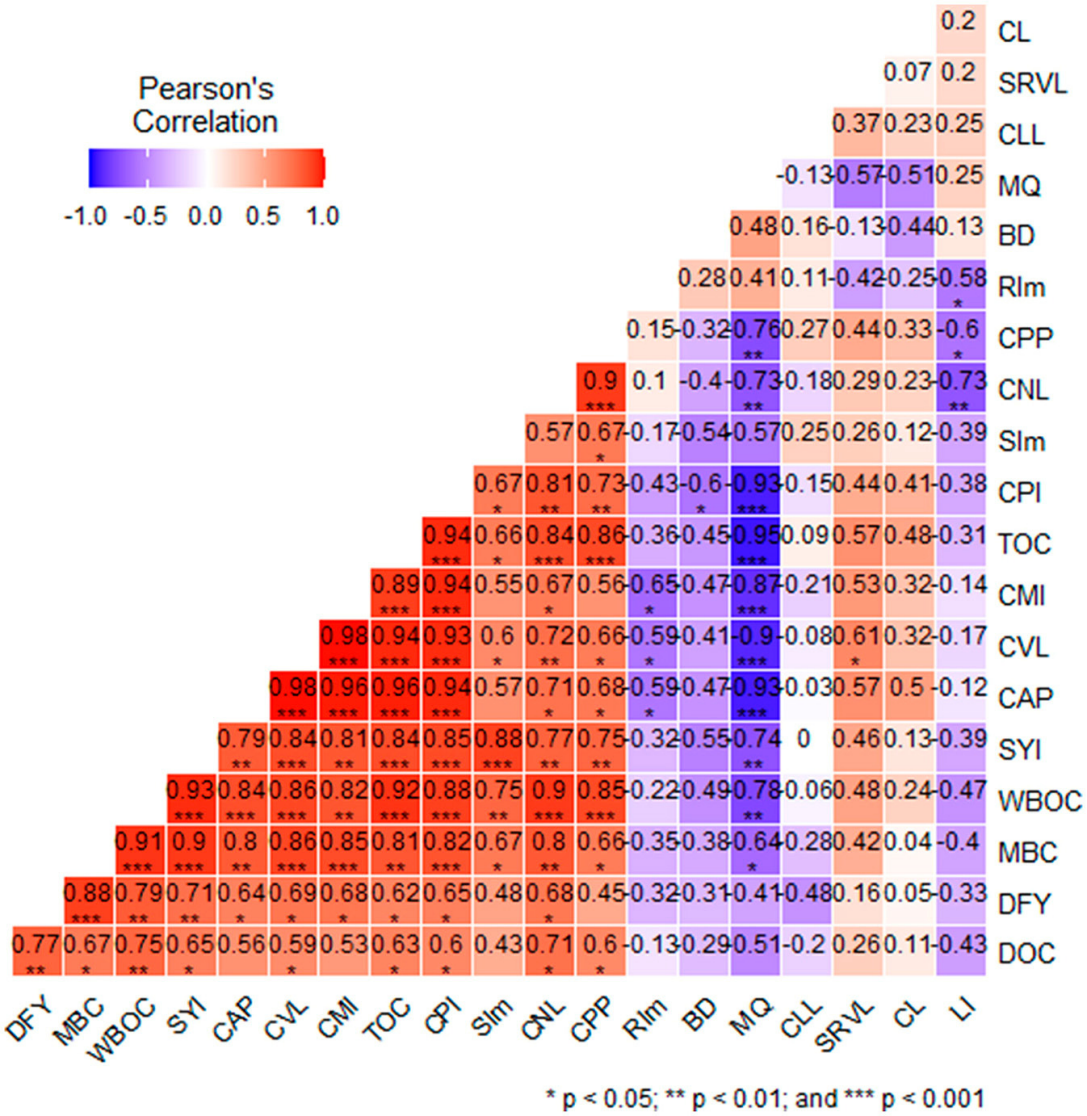
Correlation matrix among system yield attributes and SOC pools and their indices; DFY, dry forage yield; SYI, sustainable yield index; MBC, microbial biomass carbon; WBOC, Walkley–Black organic carbon; TOC, total organic carbon; DOC, dissolved organic C; RI_mean_, mean recalcitrant index; C_VL_, very labile pool of soil organic carbon; C_L_, labile pool of soil organic carbon; C_LL_, less labile pool of soil organic carbon; C_AP_, active pool of soil organic carbon; C_PP_, passive pool of soil organic carbon; RI_m_, mean recalcitrant indices; CPI, carbon pool index; CMI, carbon management index; BD, bulk density; MQ, microbial quotient; SR_VL_, stratification ratio of labile pool of soil organic carbon; SI_mean_, mean sensitivity index; without *, *, ** and *** represent non-significant, significance at *P* < 0.05, 0.01, and 0.001.

A strong positive correlation was reported among DOC, MBC, C_VL_, C_NL_, C_PP_, WBOC, and TOC (*r* ≥ 0.59^***^); these indices had a significant effect on CPI and CMI and eventually affected the DFY and SYI (Figure 2). The SI_mean_ also showed a strong positive correlation with MBC, C_VL_, WBOC, TOC, CMI, and CPI, while a strong negative correlation was observed between BD and CPI, RI_mean_ and MBC, C_AP_, C_VL_, CMI; MQ with C_VL_, C_AP_, C_NL_, WBOC, TOC, CPI, CMI, SYI; and LI with C_NL_, C_CP_, and RI_m_. Multiple regression analyses showed that WBOC and SImean explained approximately 94 and 96% variability in SYI and DFY, and MBC, DOC, and C_pp_, respectively (Table 7).

**Table 7:** Regression (backward elimination models) between soil parameters and dependent variables.

**Dependent variable**	**Intercept**	**Independent variable I**	**Independent variable II**	**Independent variable III**	***R* ^2^**	**Adjusted *R*^2^**
DFY	30.5 ± 19.0	163.3^***^ ± 24.2(MBC)	341.5^***^ ± 65.3 (DOC)	−6.15^***^ ± 1.51(C_PP_)	0.96	0.95
SYI	0.46^***^ ± 0.02	0.002^***^ ± 0.003(WBOC)	0.0004^**^ ± 0.0001(SI_m_)		0.96	0.95

^**^ and ^***^represent significance at *P* < 0.01 and 0.001; DFY, dry fodder yield; SYI, sustainable yield index; MBC, microbial biomass C; DOC, dissolved organic C; WBOC, Walkley–Black oxidizable organic C; SI_mean_, mean sensitivity index.

## 4. Discussions

Soil health and agricultural sustainability largely depend on the stock and stability of SOC in agroecosystems. Continuous cropping in semi-arid regions often decreases SOC pools, and the net depletion of TOC stock accelerates because of hot summer, perturbation of the system, and photo-oxidation of SOC (Srinivasarao et al., 2021). Soil disturbances because of tillage and other cultural practices hasten the SOC oxidation by exposing the protected C to an ultimate reduction in SOC (Six et al., 2002). Several studies reported a decline in TOC because of long-term cropping with annual crops under semi-arid and sub-tropical regions (Majumder et al., 2008b; Srinivasarao et al., 2012; Anantha et al., 2022). Contrarily, the guinea grass-based round-the-year forage production systems provided a protective covering to the soil and improved aggregation, SOC, and soil quality in pastures and grassland because of perennial growth habit, profuse rooting of the tussocks, adaptation to conservation tillage practices, and greater biomass turnover (Sanderson and Adler, 2008; Das et al., 2014; Kibet et al., 2016; Chen et al., 2022). The positive build-up of SOC in control plots in the present experiment without the application of any external carbon except through crops also supports the hypothesis of the SOC protection against oxidation loss because of the protective covering of the soil surface and reduced tillage (Rai et al., 2013; López-Vicente et al., 2021).

The long-term application of chemical fertilizer (NPK) resulted in increased SOC content because of greater return of root biomass and rhizodeposition (Srinivasarao et al., 2012; Datta et al., 2018; Pausch and Kuzyakov, 2018). With the application of FYM, the SOC pool was increased further because of the positive impact of FYM on root biomass production and its direct contribution to SOC. The integrated nutrient management under NPKF provided favorable conditions for microbial proliferation (Majumder et al., 2008a). The DOC is derived from root exudates, above-ground residues, microbial biomass debris, soluble organic carbon and liberated from decomposition of added organic matter (Christ and David, 1996; Liang et al., 2011). Therefore, NM practices NPK, FYM, and NPKF increased the DOC stock compared to the control. Earlier findings also reported higher values of DOC in NPKF-treated plots than the NPK (Benbi et al., 2015; Li et al., 2020; Mustafa et al., 2020). Approximately 90% of roots in guinea grass are distributed in the top 0–0.40 m depth (Singh, 1996). Therefore, the lowered rhizodeposition and root biomass in sub-surface soil were the reasons for decreased SOC content with increasing depth (Basak et al., 2021a; Anantha et al., 2022). The increased DOC stock in 0.3–0.7 m soil depth was mainly because of the leaching of the soluble soil organic molecules to lower depths through well-drained silty loam soil under the influence of irrigation and/or rainwater (Hussain et al., 2020).

The FYM applications rate was different for the treatment FYM and NPKF. Thus, the annual total C input to soil was greater in FYM than NPKF (*P* < 0.05). However, the content of passive pools was similar for FYM and NPKF treatments. Among the four pools (C_VL_, C_L_, C_LL_, and C_NL_), very labile and less-labile pools of SOC were sensitive to NM practices. Therefore, the C_VL_ fraction increased with annual C input. A major part of the sequestered SOC in NPK, FYM, and NPKF was retained in less labile pools (C_LL_) (Table 3). A greater amount of SOC allocation in C_LL_ with similar treatments was earlier reported for sorghum–wheat and rice–wheat cropping systems (Datta et al., 2018; Singh and Benbi, 2018; Basak et al., 2021a).

Management practices affect soil quality and influence soil functions and crop productivity. The CMI indicating higher stable SOC is used to assess the impact of management options on soil productivity. The FYM and NPKF had greater values of CMI content on imposing these practices. Greater SOC further corroborated this in less labile and non-labile pools under these treatments. The findings of this experiment were in agreement with the CMI values reported for groundnut (Anantha et al., 2022) and pearl millet–wheat systems (Moharana et al., 2012). The screening of MBC, DOC, C_pp_ for DFY and WBOC, and SI_m_ for SYI highlighted the significance of these parameters as complementary indicators for capturing the effect of nutrient management practices on soil properties and productivity potential of perennial grass–legume systems. Benbi et al. (2015) also identified MBC and DOC as the most sensitive indicator of management-induced changes in the soil.

The FYM and NPKF recorded a higher C build-up. The greater C sequestration in FYM-treated soils compared to NPK was mainly associated with a wider C: N ratio and lignin and polyphenols in FYM. These constituents of FYM form stable complexes with proteins of plant origin to protect them from further microbial attack with the resultant stabilization of SOC in soils (Majumder et al., 2008b). Earlier studies reported approximately 27% stabilization of the applied C in long-term experiments in semi-arid regions of India (Srinivasarao et al., 2012; Anantha et al., 2022). The C–stabilization vary 44–51% in FYM and NPKF treatment in perennial grass–legume cropping. The C stabilization is the outcome of the interaction of the factors *viz*., C inputs and their biochemical constituents, soil properties, soil perturbation, covering of soil surface, and net “fallowing period” in the calendar year (Schmidt et al., 2011). In this study, large biomass return, the smothering effect of component crops, and minimum soil disturbances protected the applied carbon from oxidation and were responsible for greater SOC stabilization. Lower carbon addition and increased nitrogen availability promoting organic matter decomposition in NPKF were responsible for low SOC stabilization in NPKF compared to FYM.

## 5. Conclusion

The perennial grass–legume-based cropping system favors the build-up of SOC. Nutrient management through organic manure alone, in combination with NPK fertilizers, had a greater potential to sequester carbon in the soil. Integrated nutrient management and sole FYM partitioned a greater proportion of SOC in the non-labile pool with a 44–51% stabilization of the applied carbon. FYM alone or in combination with NPK also improved the carbon management index and stratification ratio. The dissolved organic and microbial biomass C and SOC in passive pools were identified for predicting the dry fodder yield. Walkley–Black oxidizable organic C and sensitivity indices of SOC pools were good predictors for the sustainable yield index of the perennial grass–legume system. Thus, this study concludes that organics and integrated nutrient management practices can ensure the stay of the SOC in soil for a longer period. Integrating perennial grass–legume-based cropping in the farming system can also provide an opportunity to maintain long-term sustainability and offset the farm enterprises' carbon footprint in similar agro-ecologies.

## Data availability statement

The original contributions presented in the study are included in the article/Supplementary material, further inquiries can be directed to the corresponding authors.

## Author contributions

AR: conceptualization, formal analysis, funding acquisition, and original draft preparation and final editing. NB: conceptualization, formal analysis, and original draft preparation and final editing. AD: investigation and methodology. SR and JS: investigation. SD and TK: data curation and investigation. SK: investigation and funding acquisition. PC, PS, and SB: data curation and preparation of the manuscript. All authors contributed to the article and approved the submitted version.
