# Supplementary material for: Changes in soil microbial biomass and organic C pools improve the sustainability of perennial grass and legume system under organic nutrient management
Source: Front Microbiol. 2023 Apr 21;14:1173986. doi: 10.3389/fmicb.2023.1173986 (PMC10160677; doi:10.3389/fmicb.2023.1173986)
Supplement: Supplementary file 1 [file Table_1.pdf]

Title: Changes in soil microbial biomass and organic C pools improve the sustainability of perennial grass and legume system under organic nutrient management

Suppleentary Table S1. Soil organic C distribution in different pools (expressed as per cent of TOC) in experimental sites with nutrient management in soil depth

Treatment	C_{VL}	C_L	C_{LL}	C_{NL}	C_{AP}	C_{PP}
Control	35.9A	25.8A	16.8B	23.4A	61.7A	40.2B
NPK	34.7A	19.1B	23.6A	24.3A	53.9B	47.9A
FYM	39.1A	26.7A	14.4B	23.7A	65.8A	38.0B
NPKF	36.6A	28.0A	14.9B	26.5A	64.6A	41.3B
Depth (m)						
0-0.15	37.3A	14.9C	15.3B	26.6A	52.2C	41.9AB
0.15-0.30	38.6A	27.1B	15.7B	23.1A	65.7A	38.8B
0.30-0.50	36.7A	31.9A	16.9B	23.6A	68.6A	40.5AB
0.50-0.70	33.7A	25.7B	21.8A	24.6A	59.4B	46.4A
0-0.7	36.7	24.0	17.2	24.6	60.6	41.0
Treatment × Depth	ns	***	***	ns	***	ns

C_{VL}: very labile; C_L: labile; C_{LL}: less labile; C_{NL}: non–labile; C_{AP}: active; C_{PP}: passive pool of soil organic carbon; uppercase for nutrient management and lowercase for depths within a column differ significantly by Duncan's multiple range test ($P \leq 0.05$); ns and *** represents represents non-significant and significance at $P \leq 0.001$ for interaction effect (Treatment × depth) in experimental sites with nutrient management (mean over depths) and soil depth (mean over treatments)
